# 以微创胸外科为中心的肺癌综合诊疗模式

**DOI:** 10.3779/j.issn.1009-3419.2016.06.05

**Published:** 2016-06-20

**Authors:** 建行 何

**Affiliations:** 510120 广州，广州医学院第一附属医院胸外科 Department of Thoracic Surgery, the First Affiliated Hospital of Guangzhou Medical University, Guangzhou 510120, China

**Keywords:** 胸腔镜手术, 无插管麻醉, 精准医疗, Video-assisted thoracic surgery, Non-intubated anesthesia, Precision medicine

## Abstract

当今可切除肺癌的治疗已发展成为以微创外科为中心的综合治疗。微创肺癌外科不光表现为切口的缩小，更表现在切口的个体化、精细化。同时，微创外科手术的其它一系列的微创化，如麻醉的微创化（如不插管），又如裸眼3D胸腔镜为代表的手术器械的微创化、精细化与个体化等。即便是晚期肺癌的患者也因从手术中得到更多组织基因的信息而得到更精准的治疗。因此当今肺癌的治疗应该是微创胸外科为核心的综合治疗。

## Abstract (en)

The treatment for resectable lung cancer has developed to the era of comprehensive treatment based on minimally invasive surgery (MIS). MIS is not only manifested by the "shrink" of incisions, but also by the individualization and meticulous of incisions. Meanwhile, the minimal invasiveness of other procedures in MIS, such as the minimally invasive anesthesia (tubeless anesthesia) and minimally invasive, meticulous and individualized surgical instruments represented by 3D thoracoscope with naked eye. Even advanced stage lung cancer patients could receive precision treatment based on molecular information of their cancer tissue obtained by surgery. Therefore, the treatment for lung cancer should be comprehensive treatment based on MIS.

## 1 手术的整体微创方案

肺癌治疗从传统单一方法发展到现代个体化微创肺癌根治术，这是一个慢慢发展的过程，正如所谓的个体化医学，个体化外科的发展大家既期待，又迷茫。如今如何可以精确的切除病患位置，又可以做到功能的最大保护，这是我们临床大夫经常思考的问题。

在临床中，患者发现有肺癌，然后进行临床分期，可以分为两部分：可手术和不可手术。对于不可手术的患者，之后要进行组织或液体活检，得到基因类型，指导以后的治疗。可手术的患者要到外科，以前外科采用的是标准切口，所有患者都是同样的切口，但是精准医学要根据肿瘤大小确定切口大小，还需确定切口的多少，以及采用的方法。所以个体化外科首先得是个切口。手术是一个难得的打开人体的机会，所以要考虑如何使手术其不仅仅是切除的过程，还要是综合多种方法联合治疗的过程，如何把不同的方法都融合到打开切口的过程当中去，所谓的结合术中放疗、术中消融、术中冷冻、胸腔内的用药等。

以前手术中采用的是全身麻醉，如今是高选择性麻醉技术，做右侧胸腔可以只麻醉右侧胸腔，甚至于手术切口只影响两个肋间，只麻醉两个肋间。大全麻把全身从脑袋到各个器官都麻了，而高选择的麻醉肺部手术会影响躯干的神经系统，但不会影响呼吸、消化系统或泌尿等其他系统。

所以在精准医学的概念下，外科治疗会更加细化，麻醉方法细分，切口细分，切除位置细分，切除方法细分，放管方法细分，放尿管也要细分，就是所谓的越细分越精准。

在精准医学的时代，外科医生具有明显优势，因为他们可以拿到样本，然后可以进行基因检测，确定患者是否需要做辅助性治疗，若需要，采用哪种辅助治疗方法更好，哪种方法配伍更好，那种方法副作用和毒副反应更少，哪种方法性价比更好。肺癌切除入路的微创以前的切口是标准切口，后来发展为腔镜，2004年以后，发展为8种个体化切口。

我们2013年在欧洲胸心外科杂志发表了一篇文章，研究并发现微创切口比传统的开胸具有生存优势，而且并发症更少^[1]^。何教授发表文章后不到一年，*BMJ*也发表了一篇相关文章^[2]^，*Lancet Oncology*邀请我们对其进行评述^[3]^。

肺癌切除靶器官的微创切口的变化只是微创的第一步，还要考虑对靶器官的影响。周围型肿瘤以前是做肺叶切除，如今是做不同肺段切除，5种个体化微创切除方式替代单一肺叶切除。另外，根据肿瘤大小，如小于2 cm，小于1.5 cm或小于1 cm，采取不同的切除方法。对于中央型/局部晚期肿瘤，以前只有全肺切除，如今6种个体化微创切除-重建方法替代单一全肺切除。将来，亚肺叶切除、跨肺叶切除和单肺叶切除会越来越多。个体化精准切除手术，如双袖状切除术、全腔镜隆突切除重建术，只是把该切的切掉，保留肺组织。手术切除是一个很好的机会，因为把人的身体打开了，所以要将其做成很好的综合治疗，可以通过这个切口进行术后放疗，好处是低能量，而且绝对到位。术后放疗是个体化精准切除手术的有利补充。

麻醉如今医学发展很快，可以进行不插管手术，麻醉也可以进行无气管插管麻醉。我们希望将来可以像内科一样，通过静脉麻醉就可以进行肺部切除。麻醉方法从传统的全麻，发展到高选择性麻醉，现在由4种个体化麻醉方式替代单一气管插管全身麻醉。这种变化的优势是，患者在最大限度切除肿瘤以后可以快速康复。患者在术后4 h就可以下病床，还有患者在进行自主呼吸麻醉纵膈镜下气管切除重建术后3 h就可以下床行走^[4]^。

手术工具创新——裸眼3D-胸腔镜手术（video-assisted thoracic surgery, VATS）在精准医学时代，外科医师需要看的更清楚，所以开发了裸眼3D的腔镜系统，能使清晰度提高50%以上，亮度提高，更重要的是使医生没有疲劳感、头晕。我们在这个系统方面已经做了一年的研究，临床上应用也超过一年，因为可以看得更清楚可以进行很多高难度的手术，如腔镜下的隆突切除重建，腔镜下的自主呼吸麻单孔隆突切除重建术等。

## 2 精准医学

以精准医学目标要求主导的全程管理，在微创胸外科、微创根治术中，应该将这种精确的理论灌输到每个环节中，所以在每个肿瘤中心或肿瘤医院，做外科的必须建立组织库和细胞库。我们医院做了8万多份的肺癌生物标本库，10, 000多份临床的完整手术相关病历数据，之后建立肺癌术后预后的预测模型，可以预测生存，划分复发风险，指导辅助化疗等。最重要的是区分患者的复发风险，仅建议容易复发的患者做辅助治疗，避免陪绑治疗。我们先后在柳叶刀和美国肿瘤临床等杂志上发表了跨人种的术后预测模型，为这项工作奠定了理论基础^[5, 6]^。此外，还可以进行跨组学研究、代谢组学及临床应用转化。这些基础研究和临床的结合非常重要，所以需要搭建联合实验室平台。在靶点研究中检测很重要，但更重要的是时间，应该考虑如何加快检测速度。如何做到，3天或一周内就完成所有的基因检测。

## 3 总结

应将精准医学思路应用于外科及相关领域（包括围手术期管理、麻醉、手术技术等），对疾病的诊断、技术的适应症等进行细分，哪怕亚类中只有1%的患者，都筛选出来，从而真正实现个体化医疗，也就是精准医学的最高境界。
